# Pyrexia Unmasking Brugada Syndrome: A Literature Review

**DOI:** 10.7759/cureus.22489

**Published:** 2022-02-22

**Authors:** Ashish Jain, K Yagnik, Khandakar M Hussain, Sarvesh Naik, Tanvi Sharma, Asna Shahab, Muhammad Haroon Khilan

**Affiliations:** 1 Internal Medicine, Conemaugh Memorial Medical Center, Johnstown, USA; 2 Medicine and Surgery, Government Medical College, Surat, IND; 3 Medicine, School of Medical Sciences and Research, Greater Noida, IND

**Keywords:** syncope, sudden cardiac death, brs, congenital cardiac arrhythmia syndrome, brugada syndrome

## Abstract

Brugada syndrome (BrS) is an inherited arrhythmia syndrome in which asymptomatic patients tend to develop fatal arrhythmias leading to sudden cardiac death (SCD) in asymptomatic or undiagnosed cases. This review tries to shed light on pyrexia being one of the triggers to cause SCD secondary to fatal arrhythmias in patients of BrS. Pyrexia, electrolyte imbalance, alcohol intake, and drugs are common triggering factors for fatal arrhythmias in patients with BrS. Most patients are asymptomatic, while the most common form of presentation that brings the patient under medical attention is syncope or SCD. Hence, patients, especially young, who present with syncope or aborted episode of SCD with typical EKG patterns, should undergo further workup. It is essential to educate patients about the condition, possible triggers, and the importance of refraining them.

## Introduction and background

**Figure 1:**
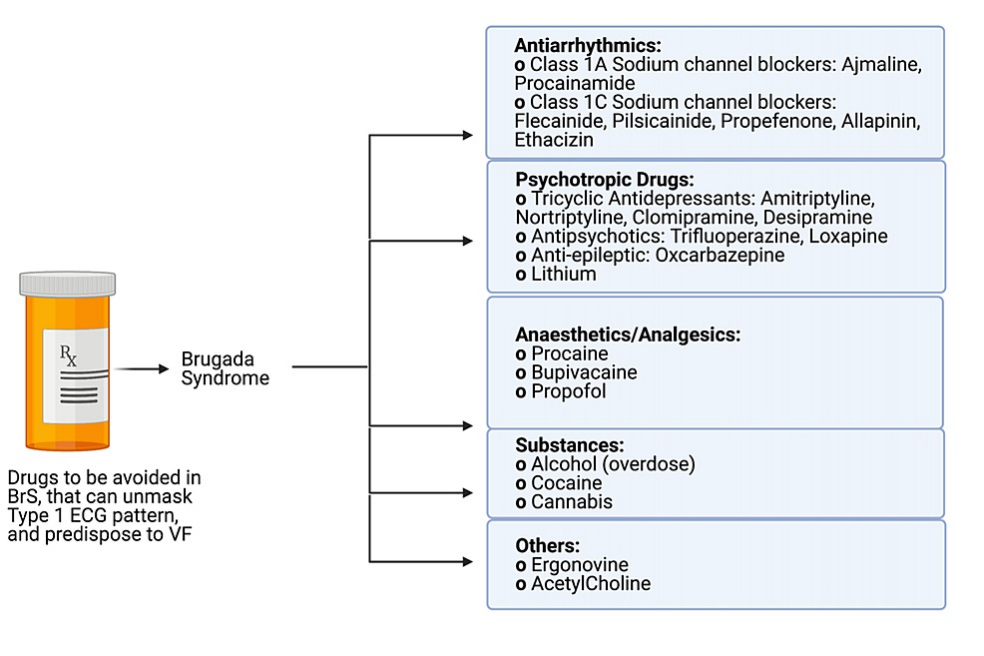
Drugs known to unmask BrS pattern on ECG and predispose to VF. Created with BioRender.com. BrS = Brugada syndrome; VF = ventricular fibrillation.

Brugada syndrome (BrS) is an inherited cardiac arrhythmia syndrome with a polygenic mode of inheritance with variable expression and many common and rare genetic variants. It causes an increased risk for ventricular tachyarrhythmia and sudden cardiac death (SCD). It is characterized by a typical ECG pattern with pseudo-right bundle branch block and persistent ST-segment elevation in the right precordial leads (V1-V3) [1], first described in 1992 by a report by two Spanish doctors "Pedro and Joseph Brugada'' who identified the pattern in eight individuals who were resuscitated from SCD caused by ventricular fibrillation (VF). Initially characterized as a right bundle branch block, persistent ST-segment elevation, and SCD syndrome, it was later renamed BrS. There is a reduction in the number or function of sodium (Na+) channels leading to a decreased sodium (Na+) current, which leads to a characteristic ECG pattern, while most of the patients stay asymptomatic. It has been shown to unmask itself in suspected individuals and is influenced by several external factors, including electrolyte imbalances, vagal tone, fever, and drugs (Figure 1). Several cases of BrS have been reported with pyrogenic conditions, which include infectious and non-infectious causes. Recently, various cases have been reported to be associated with pyrexia of coronavirus disease 2019 (COVID-19). Diagnosis of BrS is made by a typical spontaneous ECG presentation or a drug provocation test using a sodium channel blocker. The first line of management for BrS remains an implantable cardioverter-defibrillator (ICD), and other modalities such as radiofrequency ablation have shown to be effective in patients with contraindications for ICD. Asymptomatic patients or patients with ventricular premature beats or unsustained ventricular tachycardia with typical ECG findings are said to have a Brugada pattern. In contrast, patients with characteristic ECG findings who have experienced SCD or sustained ventricular tachycardia have BrS. This review focuses on the clinical presentation, diagnosis, risk-stratification, and management of pyrexia-induced BrS [2].

## Review

Clinical presentation

One-third of BrS patients are identified after evaluating symptoms such as syncope or an aborted episode of SCD [3], and spontaneous atrial fibrillation can be seen in approximately 13% of patients with BrS, suggesting an abnormality in both atria and ventricle [4]. Brugada pattern can be exhibited by a variety of diseases and conditions, including myocardial ischemia, pulmonary embolism, right ventricular compression, acute pericarditis, and electrolyte imbalances (hypokalemia, hyperkalemia, and hypercalcemia). These Brugada pattern phenotypes on ECG can result in a misdiagnosis and should be evaluated systematically, which includes electrolyte levels, cardiac markers, and imaging, such as echocardiography and CT chest with/without contrast. Pyrexia with an infectious or non-infectious etiology is a trigger known to induce fatal arrhythmias such as VF in diagnosed as well as asymptomatic/undiagnosed cases of BrS. It is critical to keep BrS as a differential diagnosis in a patient presenting with syncope, VF, or sudden cardiac arrest/death with a recent history of fever [5]. Several cases of pyrexia associated with COVID-19 have been associated with unmasking silent BrS and are mentioned below (Table 1).

**Table 1:** Recent case reports on patients of COVID-19 with BrS. COVID-19 = coronavirus disease 2019; ECG = electrocardiogram; ED = emergency department; ICD = implantable cardioverter-defibrillator; TTE = transthoracic echocardiography; VF = ventricular fibrillation; ROSC = return of spontaneous circulation; RT-PCR = reverse transcription-polymerase chain reaction; CPR = cardiopulmonary resuscitation; SpO2 = oxygen saturation; SCN5A = sodium voltage-gated channel alpha subunit 5; QTc = corrected QT; PMVT = polymorphic ventricular tachycardia.

S. No.	Authors	Study design	Country	Age of the patient	Sex	Past medical history	Presentation	ECG, angiogram, and transthoracic or esophageal echocardiography findings	COVID-19	Plan on discharge	Managed	Family history	Prior medications
1	Chang et al. [6]	Case report	USA	49	M	None	Subjective fever for one day. Syncope on the second day of fever, at rest, and regained consciousness in 1-2 min with no postictal symptoms	ECG: ST elevation. Angiogram: normal coronary arteries. TTE: no pleural or pericardial effusion, preserved cardiac function. Normal ECG after resolution of fever	Positive, by RT-PCR	Discharged on LifeVest with a plan for outpatient cardiac MRI and eventual implantation of a subcutaneous defibrillator	Paracetamol and home quarantine	History of syncope in brother	None
2	Choi et al. [7]	Case report	USA	19	M	Obesity (body mass index > 30 kg/m2) and obstructive sleep apnoea without a history of syncope or seizures	Febrile, tachycardic (117 beats/minute), hypertensive (140/91 mmHg), and hypoxemic (SpO2 94%)	Initially mild elevation of transaminases, mildly elevated ferritin level (415.6 ng/mL), and a chest X-ray demonstrating ill-defined bilateral opacities. Day 3 of hospitalization, ECG: ST-elevations (>2 mm) in leads V1 and V2 with a negative T-wave consistent	Positive, by RT-PCR	Genetic testing of the 17-gene Brugada syndrome panel showed a variant of uncertain significance in the SCN5A gene (c.4916G>A; p.G1639E), clinical status improved without subsequent Brugada pattern changes, and was discharged	Hydroxychloroquine and serial electrocardiograms to monitor QTc	He was born from a consanguineous union between parents of South American descent. Maternal uncle had epilepsy during childhood and his paternal grandfather died at an early age with unknown etiology	None
3	Kim et al. [8]	Case report	Philippines	43	M	None	Fever, nonproductive cough, myalgia, and shortness of breath for three days. Noted mild pleuritic chest pain with inspiration; however, this resolved prior to arrival in the ED	ECG was significant for 3-4-mm ST elevations, inverted T wave in leads V1 and V2. Repeat ECG showed sinus tachycardia but Brugada had resolved. Chest X-ray was significant for focal perihilar and patchy airspace opacities concerning multifocal pneumonia	Positive, by RT-PCR	Discharged with plans to follow up with cardiology as an outpatient without an implantable cardioverter-defibrillator (ICD)	Acetaminophen, intubation, and was enrolled in the remdesivir trial	No significant family history	None
4	Maglione et al. [9]	Case report	USA	58	F	Hypertension, diabetes mellitus, and Brugada syndrome, for which she underwent implantable cardioverter-defibrillator (ICD) implantation. A syncope episode was terminated by ICD shock	Multiple syncopal episodes, which were terminated by ICD shocks	Episodes of VF resolved after temperature normalized, infrequent episodes of atrial tachycardia. serial radiographs revealed the development of multifocal airspace and interstitial opacities. Sedation: unarousable. Computed tomography imaging revealed extensive intracranial hemorrhage with resultant mass effect	Positive, by RT-PCR	Irreversible neurological injury. Her family members decided to transition to comfort care.	Isoproterenol infusion (2 μg bolus followed by 1 μg/min); discontinued owing to several sustained episodes of atrial tachycardia with rates of 140-150 beats/minute, standing acetaminophen and salsalate, cooling blanket for fever control. hydroxychloroquine on hospital day one; however, discontinued the following day due to QTc prolongation to 554 ms. Broad-spectrum antibiotics remdesivir, and intubation. Anticoagulation with low-molecular-weight heparin	None	None
5	Pasquetto et al. [10]	Case report	-	52	M	None	Syncope with loss of consciousness (40 seconds) and spams occurred at bed during high fever (39.5°C), dyspnea, and fever 10 days before	ECG presented a “coved‐type” aspect in leads V1 and V2 at the fourth intercostal space and a first‐degree atrioventricular block. Computed tomography demonstrated bilateral multiple ground‐glass opacities	Positive, by RT-PCR	Genetic screening for pathogenic mutation of SCN5A. He received a subcutaneous implantable cardioverter‐defibrillator after resolution of COVID-19	Combination of high‐flow oxygen inhalation, amoxicillin/clavulanic acid, low-molecular-weight heparin, and paracetamol, with continuous ECG monitoring	None	None
6	Tsimploulis et al. [11]	Case report	USA	53	M	No significant medical history	Dry cough, myalgias, headaches, anorexia, nausea, and diarrhea for two weeks, with fevers and worsening shortness of breath for three days. Febrile to 39.9°C, hypoxic at 86% oxygen saturation on room air, and tachypneic at 32 breaths per minute, sinus tachycardia to 122 beats per minute, and blood pressure at 139/76 mm Hg	Chest radiographs showed bilateral patchy airspace opacities. Admission electrocardiogram with sinus tachycardia at 109 beats per minute, QTc of 422 ms followed by cardiac arrest from a pulseless electrical activity from hypoxia. Return of spontaneous circulation (ROSC) occurred after four minutes of cardiopulmonary resuscitation (CPR). Telemetry revealed transient sinus bradycardia with the development of a new right bundle branch block, which progressed to a junctional escape rhythm with dramatic widening of the right bundle branch block followed by the development of Brugada type I pattern and PMVT followed by cardiac arrest. After ROSC, sinus tachycardia resumed and QRS narrowed back to baseline without significant abnormalities. No QTc prolongation was noted preceding or following the event	Nasal swab - RTPCR	Continued to deteriorate with multi-organ failure. The patient’s family decided to withdraw care, and the patient immediately died after extubation	COVID-19 pneumonia with hydroxychloroquine and azithromycin. Intubation, cardiopulmonary resuscitation (CPR) using two doses of 1 mg epinephrine. Hemodynamic support with norepinephrine and epinephrine infusions propofol and dexmedetomidine infusions for sedation. ROSC - a single 200 J defibrillation, five minutes of CPR, 1 mg epinephrine. Dexmedetomidine was discontinued (contributed to his bradycardia) and his fevers were treated with intravenous acetaminophen. Four vasopressors at maximum doses with hypoxia despite maximum ventilator settings. High-dose steroids	None	None

Diagnosis

A diagnosis of BrS is based on both clinical and electrophysiological parameters. A typical ECG pattern, which was previously known as type 1 ECG with "coved ST-segment elevation" of >2 mm in the precordial leads ending with a negative T wave, is required to diagnose patients with or without symptoms. Patterns apart from the typical ECG findings of BrS cannot confirm the diagnosis and need to be further evaluated by sodium channel blocker test (flecainide (2 mg/kg over 10 minutes) and procainamide or ajmaline (1 mg/kg over 5-10 minutes)). Ajmaline has been shown to be more sensitive than flecainide or procainamide in inducing type 1 patterns [12]. A postprandial or full stomach test increases vagal tone and is another diagnostic modality that can also be used to unmask type 1 ECG patterns. The risk of conversion to ventricular arrhythmia is low if the sodium channel blocker provocation test is done under proper supervision and by experienced individuals.

Type 1 (Coved Type)

**Figure 2:**
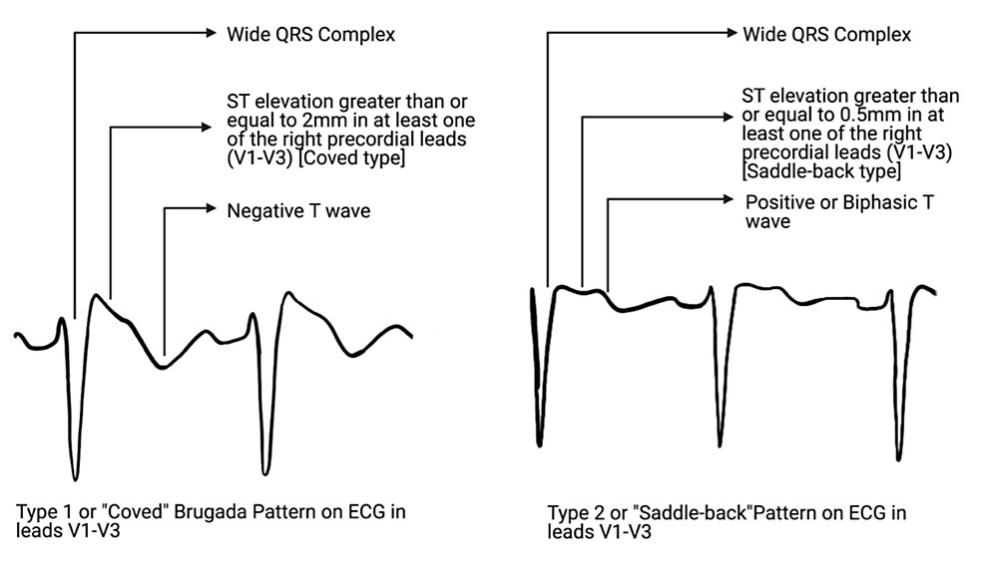
A diagrammatic representation of type 1 and type 2 Brugada pattern on EKG.

At least one of the right precordial leads (V1-V3) showing an ST elevation greater than or equal to 2 mm (J wave amplitude) gives rise to a gradually descending ST segment-terminal portion, followed by a negative T wave with little or no isoelectric separation (Figure 2) [13].

Type 2 (Saddle-Back Type)

At least one of the right precordial leads (V1-V3) showing an ST elevation greater than or equal to 2 mm (J wave amplitude) gives rise to gradually descending ST segment-terminal portion (remaining ≥1 mm above the baseline), and a subsequent positive or biphasic T wave (Figure 2) [13,14].

Type 3 (Saddle-Back Type)

At least one of the right precordial leads (V1-V3) showing an ST elevation greater than or equal to 2 mm (J wave amplitude) gives rise to descending ST segment-terminal portion (remaining <1 mm above the baseline), followed by positive T wave [13].

Genetic screening in clinical practice may be restricted to the population at risk, i.e., genetically related family members of a diagnosed patient. Over 20 genes have been identified to be associated with BrS. The sodium voltage-gated channel alpha subunit 5 (SCN5A) gene encodes the alpha subunit of the main cardiac sodium channel (Na(v)1.5), which maintains the inward sodium current and plays a vital role in regulating cardiac electrophysiological function. SCN5A gene and its more than 300 variants are associated with a quarter of cases diagnosed with BrS, these are usually loss of function variants, including non-sense variants, missense variants, nucleotide deletion/insertion variants, and splice site variants with the observation of incomplete penetrance among the carriers of the mutation.

Risk stratification

A study conducted by Priori et al. in 2013 stratified patients based on the risk of cardiac arrest, and based on their finding, patients with baseline ST elevation with syncope are the highest risk category and require an ICD placement while patients with ECG changes and no symptoms were categorized as intermediate risk and patients with ECG changes only after drug provocation were classified as low risk. The incremental predictive usefulness of an electrophysiology study (EPS) based on clinical factors is highly debatable. While some writers asserted a link between induced VF and cardiac events, extensive prospective investigations have established that an EPS does not accurately stratify the risk of arrhythmia. The most recent recommendations limit an EPS for ICD implantation to a class IIb [15,16].

**Figure 3:**
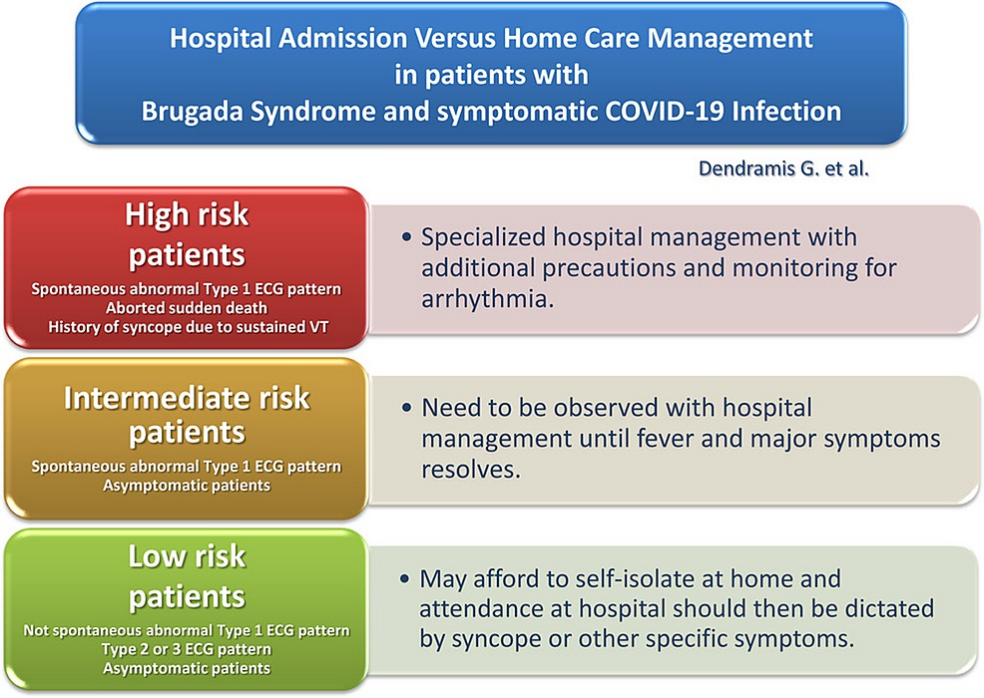
Home care versus hospital admission in patients of COVID-19 with BrS. Created with BioRender.com. BrS = Brugada syndrome; ECG = electrocardiogram; VT = ventricular tachycardia.

COVID-19 pandemic has led to hospital rush, creating a state of healthcare burnout and even collapse in some parts of the world with a shortage of primary healthcare facilities such as oxygen, medications, hospital beds, and ventilators. It is crucial to triage patients and admit only those who genuinely require expert supervision throughout their illness. Dendramis et al. gave a recommendation on hospital versus home care in patients of COVID-19 with BrS (Figure 3) [17,18].

Management

With SCN5A gene mutations, sodium channels tend to malfunction at higher temperatures, unmasking BrS; therefore, pyrexia should be treated on a priority basis, and procedures causing pain that can increase parasympathetic tone should be done under proper analgesia. The management of BrS is focused on preventing its most feared outcome, i.e., SCD in a structurally normal heart. Electrolyte imbalances such as hypokalemia, hyperkalemia, and hypocalcemia should be managed promptly, and attention should be paid while prescribing drugs known to cause ventricular arrhythmias in patients with BrS. The first step of management entails educating the patient about triggers known to induce fatal arrhythmias. The first line of management remains an ICD for symptomatic patients who have a BrS pattern on ECG and mainly type 1 history of sudden cardiac arrest/SCD or unexplained syncope. ICD has shown to be superior to both pharmacotherapy and catheter ablation. Pharmacotherapy with antiarrhythmic drugs such as quinidine or amiodarone can be tried in patients with recurrent ventricular arrhythmia requiring multiple ICD shocks or in patients who have contraindications for ICD use (refusal by the patient for ICD implantation) and significant comorbidities with a reduced life expectancy [19].

For a patient with COVID-19 with BrS, the patient should be triaged for hospital admission or home care; before discharging, pyrexia should be controlled aggressively, and hospitalized patients should be monitored for arrhythmias and hemodynamic status. Patients of COVID-19 can be dehydrated and electrolyte imbalanced due to diarrhea, vomiting, and fever; fluid management should be done conservatively, and a passive leg raise test can be used to assess preload responsiveness. In patients admitted to the ICU, checking brain natriuretic peptide (BNP) and troponin levels and performing echocardiography are recommended to assess cardiac involvement.

## Conclusions

Symptoms such as a syncopal episode with a recent history of fever could be a sign of a life-threatening underlying condition such as BrS. Pyrexia is a potential trigger for fatal cardiac arrhythmia such as VF in a previously asymptomatic patient. Taking a detailed history of previous episodes of syncope and family history of SCD plays an important role in diagnosing and risk stratifying these patients. Fever associated with the COVID-19 pandemic has unmasked various cases of BrS, and triaging patients based on their severity should be done to avoid the medical futility of critical healthcare facilities on a non-critical patient who can be managed at home.
